# The estrogen hypothesis of Schizophrenia implicates glucose metabolism: Association study in three independent samples

**DOI:** 10.1186/1471-2350-9-39

**Published:** 2008-05-06

**Authors:** Line Olsen, Thomas Hansen, Klaus D Jakobsen, Srdjan Djurovic, Ingrid Melle, Ingrid Agartz, Haakan Hall, Henrik Ullum, Sally Timm, August G Wang, Erik G Jönsson, Ole A Andreassen, Thomas Werge

**Affiliations:** 1 Research Institute of Biological Psychiatry, Sct. Hans Hospital, DK-4000 Roskilde, Denmark; 2 TOP-project, Department of Psychiatry, Ullevål University Hospital, Institute of Psychiatry, University of Oslo, Oslo, Norway; 3 Human Brain Informatics (HUBIN), Department of Clinical Neuroscience, Psychiatry Section, Karolinska Institutet and Hospital, Stockholm, Sweden; 4 Department of Clinical Immunology, Rigshospitalet, Copenhagen, Denmark; 5 University Department of Psychiatry, Frederiksberg, DK-2000 Frederiksberg, Denmark; 6 University Department of Psychiatry, Amager, DK-2300 Copenhagen S, Denmark; 7 Centre for Pharmacogenomics, University of Copenhagen, 2200 Copenhagen N, Denmark

## Abstract

**Background:**

Schizophrenia is a highly heritable complex psychiatric disorder with an underlying pathophysiology that is still not well understood. Metaanalyses of schizophrenia linkage studies indicate numerous but rather large disease-associated genomic regions, whereas accumulating gene- and protein expression studies have indicated an equally large set of candidate genes that only partially overlap linkage genes. A thorough assessment, beyond the resolution of current GWA studies, of the disease risk conferred by the numerous schizophrenia candidate genes is a daunting and presently not feasible task. We undertook these challenges by using an established clinical paradigm, the estrogen hypothesis of schizophrenia, as the criterion to select candidates among the numerous genes experimentally implicated in schizophrenia. Bioinformatic tools were used to build and priorities the signaling networks implicated by the candidate genes resulting from the estrogen selection. We identified ten candidate genes using this approach that are all active in glucose metabolism and particularly in the glycolysis. Thus, we tested the hypothesis that variants of the glycolytic genes are associated with schizophrenia or at least with gender-associated aspects of the illness.

**Results:**

We genotyped 185 SNPs in three independent case-control samples of Scandinavian origin (a total of 765 patients and 1274 control subjects). Variants of the mitogen-activated protein kinase 14 gene (MAPK14) and the phosphoenolpyruvate carboxykinase 1 (PCK1) and fructose-1,6-biphosphatase (FBP1) were nominal significantly associated with schizophrenia, and several haplotypes within enolase 2 gene (ENO2) consist of the same SNP allele having elevated risk of schizophrenia. Importantly, we find no evidence of stratification due to nationality or gender.

**Conclusion:**

Several gene variants in the Glycolysis were associated with schizophrenia in three independent samples. However, the findings are weak and not resistant to correction for multiple testing, which may indicate that they are either spurious or may relate to a particular subtype or aspect of the illness.

## Background

Schizophrenia is a severe psychiatric disorder with a strong genetic component and a complex mode of inheritance[1]. The traditional, unbiased studies of linkage analysis and positional cloning have identified a few credible risk genes of schizophrenia but have also fallen short of providing definitive results as to the actual risk alleles [2]. Although recent years have seen a growing number of association studies of candidate genes proposed to underlie possible deviant biochemical parameters of specific clinical phenotypes or deficient physiological processes in schizophrenia patients, few findings have yet been replicated [3].

A likely cause of the limited success of psychiatric genetics is the complexity of these diseases that are widely assumed to arise from an array of genetic and environmental risk factors. As a probable consequence of this complexity, the identification of genetic liability variants for schizophrenia has proven difficult and thus, the biological basis for the illness is still not well understood. Indeed, a specific clinical phenotype may well result from several risk genes affecting the same biological process as genes do not act alone but rather interact with one another or with environmental factors to modulate cellular systems[4].

The recent advance in functional-genomics studies of post mortem brains has led to new interesting hypotheses on the etiology of schizophrenia [5]. Importantly, expression studies on post-mortem human brain [6], of non-CNS tissue or body fluids [7] have been able to implicate hundreds of genes or proteins in pathology of the illness. However, these findings are likely to include a considerable number of false positives due to well-recognized caveats of post-mortem studies such as variation in the post-mortem interval, prior medication, drug abuse and age. Similarly, expression studies outside the brain will necessarily be flawed by factors that are unrelated to the schizophrenia pathology in the brain.

The challenge, therefore, is to develop a strategy that collectively interpret and employ the massive, disease-related data sets produced by unbiased (i.e. none-hypothesis driven) linkage and expression studies. This may be achieved through the use of a broadly accepted hypothesis of schizophrenia to single out genes and proteins from the unbiased experimental data sets that are more likely linked to the etiology or pathology of the disease. One compilation of widely accepted clinical and epidemiological characteristics of schizophrenia is that age of onset, symptomatology and outcome of antipsychotic treatment differ between men and women (reviewed in [8]). The estrogen hypothesis has been proposed to account for these gender differences and suggests that estrogen provides protection from the development of schizophrenia and mitigates the severity of negative symptoms[9,10]. Several clinical observations support the estrogen hypothesis. These include fluctuation of psychotic symptoms in schizophrenic women during their menstruation cycle [11-14] and indications of a higher efficacy of antipsychotic treatment in schizophrenia women than in men[15]. Finally, there are also reports on reduced levels of plasma estrogen in both male[16] and female[14,17-19] schizophrenia patients. It is known that estrogen has pleiotrophic effects on a variety of processes in the developing and adult brain [20-23], and therefore estrogen-mediated signaling cascades rather than estrogen itself are candidate pathways that may affect the risk of or modify the manifestation of schizophrenia. This implies that while estrogen varies enormously between and within gender, the putative schizophrenia risk genes downstream of estrogen need not differ between men and women.

Thus, we exploit the assumption that some risk genes of schizophrenia are subject to estrogen regulation to postulate that the most likely candidates among the unbiased, disease-related genes and proteins are those involved in estrogen mediated signaling or transcription or estrogen metabolism. Subsequently, we have grouped these estrogen-responsive and disease-related candidate genes according to their involvement in well-known cellular pathways. The best-defined candidate network that was generated contained genes that are all involved in glucose metabolism. Based on these combined lines of evidence we have tested if genetic variants of ten genes involved in glucose metabolism are associated with schizophrenia using a case-control study design on three independent samples of Scandinavian origin.

## Results

We have screened ten candidate genes due to their relationship to the estrogen hypothesis and their deregulation in – or linkage to schizophrenia. The genes were selected using an approach, which integrates data from different experimental sources as described in the material and methods section. The expression profile or linkage to schizophrenia for the ten genes is summarized in Table 2.

**Table 1:** Sample characteristics

		**Schizophrenia**		**Control**	
		
**Sample**		**Women (SD)**	**Men (SD)**	**Women (SD)**	**Men (SD)**
***Denmark***					
	N	160	224	330	478
	Mean age	45.9 (12.3)	43.6 (11.6)	45.7 (12.0)	43.3 (11.2)
	MAFA	28.4 (10.6)	26.4 (8.1)		
***Sweden***					
	N	97	160	111	182
	Mean age	57.1 (16.7)	52.3 (13.8)	50.7 (10.1)	50.1 (10.0)
	MAFA	25.9 (8.0)	23.8 (5.9)		
***Norway***					
	N	57	67	97	76
	Mean age	38.3 (11.3)	35.3 (10.3)	38.1 (10.1)	40.1 (10.1)
	MAFA	26.2 (8.7)	25.9 (9.1)		

Mean age is calculated as the expected mean age in 2006; MAFA: mean age at first admission.

**Table 2:** Reported alterations in expression pattern, linked markers and the number of 185 SNPs tested for each of the 10 candidate genes

^#^	**Gene name**	**Symbol**	**Chr**	**SNP**^1^	**Altered expressional**^2^	**Linked marker(s)**^3^	**pmid**
	**mRNA**						
1.	Enolase 2 (gamma, neuronal)	ENO2	12p13	4	Prefrontal cortex (↑)		15474909
2.	Glycogen synthase kinase 3 beta	GSK3B	3q13.3	34	Hippocampus (↓)		12363385
3.	Mitogen-activated protein kinase 14	MAPK14	6p21	40	Dorsolateral prefrontal cortex (↓)		12363385, 15098003
4.	Phosphoglycerate mutase 1 (brain)	PGAM1	10q25.3	11	Prefrontal cortex (↓)		11576761
							
	**PROTEIN**						
5.	Enolase 1, (alpha)	ENO1	1p36.2	5	Prefrontal cortex (↓)		15098003
1.	Enolase 2 (gamma, neuronal)	ENO2	12p13	-	Prefrontal cortex (↓)		15098003
6.	Glyceraldehyde-3-phosphate dehydrogenase	GAPDH	12p13	3	Prefrontal cortex (↓)		15098003
2.	Glycogen synthase kinase 3 beta	GSK3B	3q13.3	-	Prefrontal cortex (↓), frontal cortex (↓)		15098003, 11290401
7.	Hexokinase 1	HK1	10q22	32	Prefrontal cortex (↑, ↓)		10784483, 15098003
4.	Phosphoglycerate mutase 1 (brain)	PGAM1	10q25.3	-	Prefrontal cortex (↓)		15098003
							
	**LINKAGE**						
4.	Phosphoglycerate mutase 1 (brain)	PGAM1	10q25.3	-		D10S677	9754621
8.	Phosphoenolpyruvate carboxykinase 2	PCK2	14q31.1	4		D14S70	12808429
9.	Phosphoenolpyruvate carboxykinase 1	PCK1	20q13.31	8		D20S100	11803533
10.	Fructose-1,6-bisphosphatase 1	FBP1	9q21.33	15		D9S1812	12808429
3.	Mitogen-activated protein kinase 14	MAPK14	6p21	-		D6S1610, D6S271,	12929083,11126394,
						D6S291, HLADQA,	12140777,7581457
						HLADQB, HLA-DQB1,	
						HLADRB1, D6S273,	
						HLAA, D6S464	

^1 ^The number of heterogeneous SNPs investigated for each gene in the current study

^2 ^Tissue and the direction of expression alterations in schizophrenia as compared to control subjects. ↓: Reduced expression, ↑: Elevated expression

^3 ^Markers with suggestive evidence for linkage to schizophrenia in the vicinity of the given gene

### Single SNP association analysis

Allele frequencies between the case and control groups were compared and adjusted for stratification due to nationality and gender, respectively. Among the 156 analyzed SNPs, five SNPs within three genes obtained nominal P-values (< 0.05) when adjusting for nationality (Table 3 & Additional file 1). Three of these five SNPs reside within the MAPK14 gene. Although, the minor allele frequencies of these SNPs are low their odds ratios point in the same direction and suggest that variants of MAPK14 could provide protection against schizophrenia. The C alleles of both rs4129219 and rs1040566 in each of the FBP1 and the PCK1 gene showed nominal association with elevated risk for schizophrenia (Table 3). There was no significant evidence for heterogeneity of the odds ratio between countries for these five SNPs (Table 3). The results were similar when we adjusted for gender as the odds ratios and the corresponding p-values were only marginally altered as compared to the results above (Table 3). Furthermore, we found no evidence for gender stratification as reflected in the non-significant outcome of the Breslow-Day test (Table 3).

**Table 3:** Minor allele frequency and associated p-values for markers showing nominal significant allelic effect

					**Allelic association adjusted for**			
					
			**Minor allele frequency**		**Nationality**			**Gender**
			
**Gene**	**Marker**	**Variants**^1^	**Cases**	**Controls**	**OR**	**CI (95%)**	**P_nat _(P_BD_)^2^**	**P_sex _(P_BD_)^3^**
MAPK14	rs9470207	C/T	0.01	0.02	0.52	(0.30–0.92)	0.02 (0.25)	0.02 (0.97)
MAPK14	rs6908372	T/A	0.01	0.02	0.54	(0.31–0.96)	0.03 (0.25)	0.03 (0.93)
MAPK14	rs9462156	A/G	0.01	0.02	0.56	(0.32–1.00)	0.04 (0.36)	0.04 (0.91)
PCK1	rs1040566	C/A	0.11	0.09	1.27	(1.02–1.59)	0.04 (0.82)	0.05 (0.23)
FBP1	rs4129219	C/T	0.19	0.17	1.20	(1.01–1.43)	0.04 (0.92)	0.09 (0.45)

OR: odds ratio, CI(95%): 95% confidence interval for the OR

^1 ^The minor allele is give prior to the major allele

^2^P-values are for the Cochrane-Mantel-Haenszel tests adjusted for potential presence of stratification between countries. P-values for the Breslow-Day test statistics for odds ratio heterogeneity between countries are given in ()

^3^P-values are for the Cochrane-Mantel-Haenszel tests adjusted for potential presence of stratification between men and women in all 3 countries. P-values for the Breslow-Day test statistics for OR heterogeneity between gender are given in ()

### Haplotype association analysis

Haplotype analyses were conducted for SNPs within LD blocks of each gene individually. Only haplotypes within one gene (ENO2) showed a nominal significant effect (Table 4). We did not find evidence of stratification due to gender or the presence of different nationalities in the sample. Although, none of the SNPs within the ENO2 haplotypes were significantly associated in the single locus allele tests one four-marker ENO2 haplotype (A-T-A-T of rs2238116, rs1057077, rs710415, rs3213434, respectively) showed significant association (Table 4). As can be seen in Table 4 all of the two and three marker combinations within this haplotype, which includes the T allele of the rs3213434 locus, also had significant p-values.

**Table 4:** ENO2 haplotypes showing nominal significant association to schizophrenia

	**Model effect**^1^		**Individual effect**			
	
	**Overall Country**		**Case**	**Control**		
**Markers**	**P**	**P**	**n (freq)**	**n (freq)**	**OR (CI: 95%)**	**P**^2^
**rs2238116, rs3213434**	0.01	0.59				
AC*			1030 (0.72)	1730 (0.71)	1.00 (1.00-1.00)	0.51
**AT**			20 (0.01)	69 (0.03)	0.48 (0.29–0.80)	0.0007
GT			388 (0.27)	655 (0.27)	1.00 (0.86–1.17)	0.74
**rs1057077, rs3213434**	0.0026	0.32				
TC*			1087 (0.72)	1782 (0.70)	1.00 (1.00-1.00)	0.33
**TT**			18 (0.01)	69 (0.03)	0.42 (0.35–0.51)	0.0006
AT			403 (0.27)	679 (0.27)	0.99 (0.58–1.69)	0.92
**rs710415, rs3213434**	0.01	0.37				
AC*			1091 (0.72)	1772 (0.71)	1.00 (1.00-1.00)	0.31
**AT**			18 (0.01)	63 (0.03)	0.46 (0.27–0.79)	0.0009
GT			399 (0.26)	675 (0.27)	0.97 (0.84–1.13)	0.92
**rs2238116, rs1057077, rs3213434**	0.0045	0.59				
ATC*			1021 (0.72)	1725 (0.71)	1.00 (1.00-1.00)	0.57
**ATT**			17 (0.01)	66 (0.03)	0.43 (0.36–0.52)	0.001
GAT			388 (0.27)	653 (0.27)	1.01 (0.83–1.23)	0.71
**rs2238116, rs710415, rs3213434**	0.01	0.61				
AAC*			1025 (0.72)	1712 (0.71)	1.00 (1.00-1.00)	0.63
**AAT**			17 (0.01)	61 (0.03)	0.47 (0.27–0.81)	0.001
GGT			386 (0.27)	643 (0.27)	1.01 (0.87–1.18)	0.67
**rs1057077, rs710415, rs3213434**	0.01	0.32				
TAC*			1085 (0.72)	1765 (0.71)	1.00 (1.00-1.00)	0.50
**TAT**			18 (0.01)	63 (0.03)	0.47 (0.47–0.47)	0.004
AGT			399 (0.27)	668 (0.27)	0.99 (0.99-0.99)	0.95
**rs2238116, rs1057077, rs710415,**	0.01	0.60				
**rs3213434**						
ATAC*			1019 (0.72)	1710 (0.71)	1.00 (1.00-1.00)	0.68
**ATAT**			17 (0.01)	61 (0.03)	0.47 (0.39–0.57)	0.004
GAGT			386 (0.27)	643 (0.27)	1.02 (0.84–1.24)	0.70

*Reference haplotype with odds ratio (OR) set to 1.00

^1 ^P-values for the overall effect of a model containing the given loci and the presence of stratification due to differences between the 3 countries.

^2 ^P-value for the individual haplotype adjusted for nationality

The rs6908372 marker of MAPK14, which showed single marker association, falls in a LD block that contains one additional tagSNP (rs851017). The haplotype analysis of these two loci showed a borderline effect (P = 0.07) with an estimated haplotype frequency of the significant allele (T-C) of 0.01 and 0.02 in cases and controls, respectively and an odds ratio of 0.58 (CI (95%): 0.33–1.01, P = 0.04).

The remaining four of the five SNPs, which showed single marker association for MAPK14, FBP1 and PCK1 are single tagSNPs for separate LD blocks (i.e. we have no data for other gene variants within these blocks).

### Analysis of pair wise SNP interactions

PLINK was employed for the analysis of epistatic effects of genetic variants among the nine SNPs, which showed nominal association signals in either the allele or the haplotype test. However, we found no significant differences in any of the pair wise odds ratios between cases and controls suggesting that there are no interactions among these nine markers (data not shown).

### Combined analysis across genes

Two statistical strategies were applied in order to attempt to establish the study hypothesis by combining evidence across genes. First, we asked whether the number of nominally significant genes were higher than expected by chance. The total number genes nominally associated with schizophrenia (four out of the ten genes examined: three genes by the single marker analyses and one gene by the haplotype analyses) was compared to that expected by change at the 0.05 significance level. This is a significant excess of what would be expected by chance (p < 0.001) and argues in favor of the overall hypothesis that glucose metabolism is implicated in schizophrenia. Second, we applied the truncated product p-value method that takes all computed p-values into account [Zaykin et al., 2002]. This analysis did not, however, provide support for the study hypothesis in total or gene-wise (p > 0.33).

### Formal correction for multiple testing

A conservative correction for multiple testing of the single locus analysis would imply considering all 156 SNPs that have been assayed. However, the 156 analyses are not independent for two reasons. Firstly, they do not address 156 different hypotheses but rather ten, one hypothesis for each gene selected in the study. Secondly, the 156 SNPs are not independent, many of them being in high LD. Thus, the least strict correction would be by the number of genes, i.e. by a factor of ten. In either case none of the single locus findings survive correction. A similar rational could be applied to the haplotype analysis. None of these resist correction for the number of SNPs or the number of haploblocks analyzed. However, the haplotype findings do seem to oppose the much less restrictive correction by the number of examined genes.

## Discussion

In this study we present a novel method to select unbiased candidate genes for association studies. We use experimental data from schizophrenia studies on gene expression and linkage analysis – i.e. techniques that are generally hypothesis-free approaches – and combine them with the well-founded clinical and epidemiological hypothesis of neuroprotective effect of estrogen. The hypothesis serves as a clinical or biological filter that reduces the noise in the experimental data sets by eliminating genes that were found by chance in the studies on gene expression or linkage analysis. As the final selection criteria, we only consider genes that are classified as constituting a functional signaling network. Using this stepwise approach we found that the glycolysis is a plausible candidate network of schizophrenia, being compatible with both unbiased experimental data and with one of the most well established hypothesis of the disease. Importantly, the brain relies almost exclusively on glucose for its source of energy and the coordinated activity of the glycolytic processes are mandatory for proper brain development and function. Nonetheless, the functional relatedness between the glycolytic genes in the unbiased data sets has not been realized earlier. Four aspects emerge from the study that merit particular commenting.

Firstly, it is important to emphasize that the selection procedure chosen in this study – albeit based on data from unbiased studies on gene expression and linkage analysis – is itself biased by the choice of clinical hypothesis that is used to filter the experimental input data and by the current knowledge on biological networks that is used to recognize and prioritize the identified gene clusters. However, we consider this type of bias to be a positive feature of our selection method that fundamentally allows any well-formulated hypothesis to be tested against the collection of unbiased experimental data available at any given time.

Secondly, the study focused on genes that affect glucose metabolism and their involvement in schizophrenia and we have completed an association screen of ten candidate genes for schizophrenia in a large Scandinavian sample. We aimed to provide high coverage of each gene by selecting (i) a set of tagSNPs that characterized common variation across each gene or (ii) as many of the known SNPs with a maf > 0.01 in Caucasians. We have highlighted the most promising single- and multi marker associations on the basis of nominal P-values.

In the single marker analyses we identified three genes containing one or more SNPs with a nominal significant effect and multi marker association analysis identified one additional gene (ENO2). All five SNPs, which showed single locus association to schizophrenia, are located in introns and have been selected as tagSNPs for LD blocks. They may therefore not represent causative SNPs but rather be in LD with true disease susceptibility variants. Although the allele frequencies for the risk variants of rs4129219 (FBP1) and rs1040566 (PCK1) are somewhat larger than those observed for the 3 MAPK14 SNPs the odds ratios (OR: 1.20 and 1.27 for FBP1 and PCK1, respectively) are relatively low even for complex conditions.

Multi marker analysis revealed that the significant ENO2 haplotypes all includes the T allele of the rs3213434 locus although the single locus allelic test of this polymorphism is not significant on it's own. This suggests that the significant ENO2 haplotype(s) tag a true causative associated variant(s), which seem to have originated on a haplotype background of carriers of the rs3213434 T allele. The statistics for combined effect of the two MAPK14 markers is uncertain, as the confidence interval for the significant T-C haplotype is fairly broad. Also, P-values for all five single markers as well as for the ENO2 haplotypes are non-significant if adjusted for multiple testing.

One main reason for the difficulty in identifying significant risk alleles is presumably small effect sizes. In fact, this study was able with a power = 0.8 to detect odds ratios larger than 2.8 for the SNP with the minimal MAF (= 1%) and ORs larger than 1.28 for the SNP with the maximal MAF (= 49%) analyzed in this study (Supplementary Table 1). The possibility also exists that the disease susceptibility genes are not shared among all individuals thus making them harder to identify. Furthermore, several risk genes may interact and thereby jointly increase the overall risk for developing schizophrenia. Ideally, interaction analyses between genes should therefore be performed to address this complexity. However, this provides additional multiple testing problems to that of testing many gene variants in several (or many) genes. As our set up invites for combined analyses of the different genes we performed post hoc interaction analyses among the nine SNPs, which showed nominal association signals in either the allele or the haplotype test but found no evidence for such interactions. Although the presences of interactions between other markers are overlooked we restricted the analysis to the nine markers to limit the number of tests to be performed.

Thirdly, gender differences are well-established in schizophrenia and the immense literature on the subject emphases the importance of taking these differences into account when searching for biological susceptibility factor for this disorder[24]. The estrogen hypothesis – formulated to account for these differences – suggests that the observed symptom variability between men and women (e.g. in age of onset, affective and flat features) is related to estrogen. However, estrogen may not be directly involved in schizophrenia pathogenesis. Rather, we have based the current study on the assumption that schizophrenia susceptibility genes are regulated by estrogen. The rationale behind this assumption rests on the growing evidence that estrogen affects a variety of processes in the developing and adult brain, including neuronal differentiation, survival and excitability (reviewed in[25,26]) as well as on glial proliferation and synaptic plasticity (Reviewed by[22,27]). Also, estrogen is known from animal studies to regulate several neurotransmitter systems[22,28] (dopamine, serotonin, noradrenalin and glutamate) implicated in schizophrenia.

We find no significant gender differences in the SNPs related to glucose metabolism, selected because of their compatibility with the estrogen hypothesis. This may at a first glance seem unexpected. However, the strategy in this study is not based on the assumption that estrogen itself is a risk factor of schizophrenia, but rather on the clinical and epidemiological observations that indicate that estrogen affects liability or the clinical manifestation of the disease. If this assumption is true, then the estrogen-signaling network is functionally coupled to the cellular or physiological processes that predispose to schizophrenia that we attempt to identify. Importantly, the risk associated with disease-disposing alleles downstream of estrogen need not differ between men and women, while the clinical estrogen-effects do fluctuate as a consequence of the inherent gender-related variations in the metabolism of estrogen[14,29-31].

Finally, to our knowledge there have been no previous studies reporting association between the glycolytic genes investigated in the present study and schizophrenia. As we cannot draw firm conclusions with the current data further investigations with testing of more loci are required to clarify whether ENO2, FBP1, MAPK14 and PCK1 variants provide genetic support for the view that alterations in glucose metabolism are intrinsic to schizophrenia etiology.

We only examined genes in the glycolysis that are also subject to estrogen regulation. Thus, other genes involved in glucose regulation that could be implicated in schizophrenia, were not included in this study due to our sampling scheme for candidate genes. Indeed, Stone et al[32] have found linkage between another glycolytic gene (6-phosphofructose-2-kinase) and schizophrenia in a mixed sample of African and European Americans. This finding has not been replicated in other samples. Both the Stone study and ours encourage to pursue the hypothesis that glucose metabolism is involved in schizophrenia.

Co-morbidity between schizophrenia and diabetes has been known for decades[33] and reports on abnormal glucose regulation go back more than half a century (e.g.[34,35]). However, the involvement of abnormal glucose regulation in the pathology and etiology of schizophrenia has not received much attention until obesity, hyperglycemic and hyperlipidemia effects was found to be associated with some of the newer, atypical antipsychotic drugs[36,37]. These observations have stimulated speculations that some antipsychotic drugs accelerate pre-existing perturbation of glucose metabolism in schizophrenic patients and thereby reveal an otherwise hidden biological basis for the disorders[38]. Indeed, a recent study by Bahn and colleagues found that elevated glucose levels in cerebrospinal fluid in drug-naïve patients were normalized upon antipsychotic medication[39]. This and other studies by the same group seem to suggest that compromised energy metabolism in particular in response to oxidative stress is intrinsic to schizophrenia [40-42]. Interestingly, oxidative stress itself has recently been linked to schizophrenia[43] possibly hinting at a disease-susceptible regulatory network in the brain.

## Conclusion

In conclusion, we failed to provide convincing evidence in support of the hypothesis that genetic variations in the investigated glycolytic genes are involved in the etiology of schizophrenia. Indeed, the data do encourage continued investigations on the role of glucose metabolism in this disorder. Especially, more data on the genes, which have shown nominal effects in this and other studies as well as other glycolytic genes, are needed to evaluate if dysregulation of glucose metabolism is inherent to schizophrenia.

## Methods

### 2.1 Strategy for candidate gene selection

The candidate genes that are analyzed in this study are involved in a well-defined cellular network and have been selected using a three-step strategy, which integrates functional genomics data, findings from linkage analyses and a well-founded clinical and epidemiological hypothesis.

***Step 1. ***Genes were selected according to the following two criteria: (I) they have been shown in previous studies to be differentially expressed (at the mRNA and/or protein level) in brain tissues of schizophrenia patients as compared to non-psychiatric control subjects, or (II) they are localized within +/- 1 mb of a genetic marker that have shown suggestive evidence for linkage to schizophrenia (LOD-score ≥ 2). ***Step 2. ***Genes or proteins involved either in the estrogen metabolism or are regulated by estrogen were selected among the genes identified in step 1. ***Step 3. ***The cellular signaling networks that are represented by genes or proteins selected in step 2 were identified and prioritized. The top-ranking network was selected for genotyping analysis.

#### Literature search

A systematic search in PUBMED (release April 16. 2006) for papers on schizophrenia resulted in 241 references covering three experimental areas: gene-(113) and protein (98) expression and linkage (35) studies. We only considered data represented with statistical support in the original paper that were altered at the mRNA and/or protein level in schizophrenia brain samples – and/or potentially associated with schizophrenia due to their genomic position close to markers with suggestive linkage with schizophrenia.

#### Gene and protein expression data on schizophrenia (step 1a)

The gene expression data include reports from micro-array, RT qPCR, in situ hybridization, and Northern Blotting studies. Data for protein expression was collected from studies based on immunohistochemistry, Western Blotting, ELISA and 2D gel electrophoresis. In total, we identified 339 genes and 115 proteins that have been reported with differential brain expression in patients with schizophrenia as compared to non-psychiatric control subjects, regardless of tissue specific expression.

#### Linkage data on schizophrenia (step 1b)

Data on genetic markers showing linkage to schizophrenia were collected from linkage studies based on extended family- or affected sib pair methods. Genes situated within nearby genomic regions of each genetic marker were extracted from the Ensemble genome database (release 38). Overall, 134 markers were reported having suggestive evidence for linkage (LOD score 2) to schizophrenia in at least one study. A total of 1339 protein coding genes are located within +/- 1 mb of these markers.

#### Estrogen responsive genes (step 2)

Information on genes that are regulated by estrogen were obtained from the Dragon Estrogen Responsive Genes Database (ERGDB)[44] and genes involved in estrogen metabolism, signaling or transcription were identified from public literature databases using two literature mining tools: Information Hyperlinked over Proteins (iHOP)[45] and Pathway assist[46]. A total of 2239 estrogen responsive genes were identified. Among these, 302 genes were identified among the 1751 disease-related genes identified in step 1a & b (above); 148 of the 302 genes were implicated by gene (111) or protein (53) expression studies, whereas 154 genes came from linkage studies. Only eight of the 302 estrogen-selected genes were implicated by both expression and by linkage studies. No single gene was implicated in all three criteria gene expression, protein expression and linkage data.

#### Selection of candidate genes from biological networks (step 3)

The final selection of candidate genes was performed by first identifying and prioritizing the biological networks represented by the 302 genes resulting from step 2. To identify these biological networks we have used the Database for Annotation, Visualization, and Integrated Discovery (DAVID v. beta2)[47]. DAVID is a tool for construction of functional networks among genes based on Gene Ontology (GO), on protein structure databases and on the Kyoto Encyclopaedia of Genes and Genomes (KEGG)[48]. Using the options for "Gene Functional Classification" and "medium stringency" 13 groups were constructed and prioritized according to their Group Enrichment Score that ranks the biological significance of gene groups based on overall EASE scores of all enriched annotation terms[47].

Each group of genes was also evaluated manually. The seven lowest ranking groups contained proteins performing similar, general functions (e.g. transporters, receptors, kinases, transcription factors), but which otherwise had no direct cellular relations. Another five clusters were loosely defined through their association to general molecular mechanisms that did not constitute a formal signaling pathway or network (e.g. macromolecular metabolism or cellular defense) and were also disregarded in the present study. In contrast, the remaining cluster contained five genes (i.e. Enolase 1 [ENO1], Enolase 2 [ENO2], Glyceraldehyde-3-phosphate dehydrogenase [GAPDH], Hexokinase 1 [HK1] and Phosphoglycerate mutase 1 [PGAM1]) that all participate in a single and well-defined cellular process – namely the glycolysis – and were therefore selected as a "genuine" biological network for further analysis.

**Figure 1:**
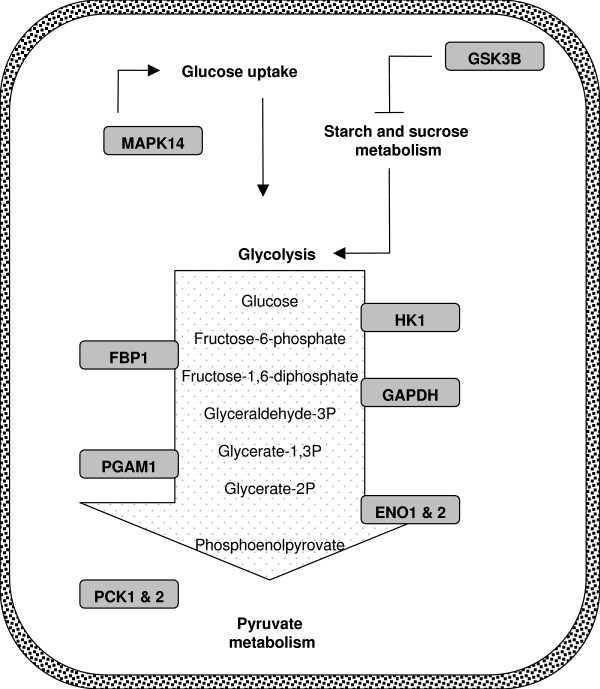
**Overview of the ten identified candidate genes and their involvement in glucose metabolism**. The glucolysis is shown as a white vertical arrow in which the relevant substrates & products are listed. The ten candidate genes are shown in rounded boxes.

Another two genes were also chosen for analysis although they were classified into the second-ranked group of "macromolecular metabolism" since they are important for regulation of glucose metabolism (i.e. Glycogen synthase kinase 3 beta (GSK3B) and Mitogen-activated protein kinase 14 (MAPK14)). Furthermore, inspection of an extended data set from the linkage analysis studies that contained genes within +/- 10 mb of a genetic marker showing association to schizophrenia (with a LOD score ≥ 2) revealed three additional genes involved in sugar homeostasis (Phosphoenolpyruvate carboxykinase 1 (PCK1), Phosphoenolpyruvate carboxykinase 2 (PCK2) and Fructose-1,6-bisphosphatase 1 (FBP1)). These three genes were also included into the final data set of 10 candidate genes. Figure 1 gives an overview of the involvement in glucose metabolism for these genes. Finally, the relationship between the selected disease candidate genes and estrogen were confirmed by manual inspection of the relevant literature.

#### Data storage

The individual gene or protein information was systematically stored in a MySQL relational database. To facilitate integration of data from the numerous studies the official HGNC nomenclature (Aug 2005 update) for gene names and symbols were obtained for each gene or the gene coding for a given protein.

### 2.2 Sample

This study included a Scandinavian case-control sample composed of three independent population-based samples of Danish, Swedish and Norwegian origin from which recent non-European ancestry had been excluded. Overall the sample includes 765 cases and 1274 control subjects. The characteristics for the three samples are summarized in Table 1.

#### The Danish sample

The Danish sample included 384 patients who have been recruited to Danish Psychiatric Biobank from the psychiatric department at the six hospitals in the Copenhagen region. All patients had been clinically diagnosed with schizophrenia according to ICD-10; F20 (n = 345), F21 (n = 6), F22 (n = 5) and F25 (n = 28) without ever having received a diagnosis of mania or bipolar illness (F30-31). An experienced research- and consultant psychiatrist verified high reliability of the clinical diagnoses [49,50] using OPCRIT semi-structured interviews. The vast majority of the patients (96%) who fulfilled the ICD-10 criteria of schizophrenia also complied with the corresponding DSM-IV standards. At the time of the last assessment the patients had a mean age of 44.5 (+/- 12.0) years, a mean age at onset of illness of 27.1 (+/- 11.2) years, a mean duration of illness of 17.3 (+/- 10.4) and a mean duration of hospitalization of 12.8 (+/- 8.6) years. None of these variables differed significantly between men and women. The majority (87%) of the patients were ethnical Danish, i.e. the patients and both parents were born in Denmark, while in a minor fraction of the cases (13%) one Caucasian parent was born outside Denmark in another North-Western European country, primarily in Sweden or Norway, secondarily in Germany, the Netherlands, England or France.

The healthy controls subjects were anonymously selected among 72,000 unpaid voluntary blood-donors from the Blood Donor Corps in the Copenhagen area. The Donor corps includes 4.9% of the adult population aged 18–65 years. Apparent behavioral abnormality was an exclusion criterion and all individuals stated that they felt completely healthy with a possibility to discuss any health related issues with a physician. Each patient was matched to on gender, year of birth and month of birth to two unrelated healthy control subjects of equivalent ethnic background.

The Danish Scientific Committees (J.nr. 01-024/01) and the Danish Data Protection Agency (J.nr. 2001-54-0798) approved the study and all the patients have give written informed consent prior to inclusion into the project.

#### The Swedish sample

The Swedish sample included 257 patients who have been recruited from psychiatric clinics in northwestern Stockholm County. All patients had been clinically diagnosed with schizophrenia (n = 224), schizophreniform disorder (n = 8) or schizoaffective disorder (n = 25) according to DSM-III-R/DSM-IV diagnostic criteria based on interviews and record reviews as previously described. [51-53]

At the time of the last assessment the patients had a mean age of 43.6 (+/- 14.2) years, a mean age at onset of illness of 24.6 (+/- 6.8) years, and a mean duration of illness of 19.0 (+/- 13.8) years. Psychotic women were older when entering the study (46.9 +/-15.5 years), and tended to have a higher age at onset (25.8 +/- 8.0 years) and a longer duration of illness (21.0 +/- 14.7 years) than men with psychosis (41.6 +/- 13.1, 23.9 +/- 5.9 and 17.7 +/- 13.2 years; Wilcoxon/Kruskal Wallis tests p = 0.007, p = 0.07, and p = 0.09, respectively). All patients were Caucasian. Based on the birth country of the grandparents or greater grandparents, 78%, 12% and 9% of the patients were estimated to be of Swedish, Finnish or other European origin, respectively. The healthy controls subjects were recruited among subjects previous participating in biological psychiatric research at the Karolinska Institute or drawn from a representative register of the population in Stockholm County and interviewed as previously described [54]. All controls were Caucasian and 86%, 6%, and 8% were estimated to be of Swedish, Finnish or other European origin, respectively. The mean age of the participants when entering the study was 40.5 (+/- 9.8) years. None of the controls suffered from schizophrenia.

The Ethical Committee of the Karolinska Hospital, the Stockholm Regional Ethical Committee and the Swedish Data Inspection Board approved the study. All subjects participated after giving informed consent.

#### The Norwegian sample

The Norwegian sample included 124 patients who had been recruited to the TOP study from all the psychiatric hospitals in the Oslo area. The patients had been clinically diagnosed according to Structural Clinical Interview for DSM-IV (SCID) with schizophrenia (n = 99), schizoaffective (n = 19), and schizophreniform disorder (n = 6). Two clinical professors continuously trained and supervised a group of research fellows in order to secure the quality of the clinical assessments. Reliability of the clinical diagnosis has recently been tested, and the percentage of agreement was 82%, and Kappa 0.77 (95% CI: 0,60–0,94).

At the time of the last assessment the patients had a mean age of 36.6 (+/- 10.9) years, a mean age at onset of illness of 26.0 (+/- 9.0) years (onset defined as age of first contact with psychiatric health service), and a mean duration of illness of 10.6 (+/- 10.5) years. None of these variables differed significantly between men and women. The majority (90%) of the patients were ethnical Norwegian, i.e. the patient and both parents were born in Norway, while in a minor fraction of the cases (10%) one parent was born outside Norway in another North-western European country.

The healthy controls subjects were randomly selected from statistical records of persons from the same catchments area as the patient groups. Only subjects born in Norway were contacted by letter and invited to participate. All controls were of Caucasian origin; around 85 % had two Norwegian parents, the rest one parent from other European origin. Moreover, all participants had to have Norwegian as their first language or have received their compulsory schooling in Norway. The mean age of the control subjects in 2006 was 39.0 (+/- 10.2) years. The control subjects were screened by interview and with the Primary Care Evaluation of Mental Disorders (PRIME-MD). None of the control subjects had a history of moderate/severe head injury, neurological disorder, mental retardation or an age outside the age range of 18–60 years. Healthy subjects were excluded if they or any of their close relatives had a lifetime history of a severe psychiatric disorder (schizophrenia, bipolar disorder and major depression), a history of medical problems thought to interfere with brain function (hypothyroidism, uncontrolled hypertension and diabetes), or substance abuse.

The Norwegian Scientific-Ethical Committees (approval no 493-03-01179) and the Norwegian Data Protection Agency (approval no 2003/2052) approved the study and all patients have given written informed consent prior to inclusion into the project.

### 2.3 SNP selection

For each gene (including 500–2000 bp upstream of exon1) TagSNPs were selected (R^2^>0.8) from genotype data (allele frequency >.01) on the HapMap European (CEU) sample (Build 35) using Tagger[55] implemented in Haploview 3.2 [56] with the options for aggressive 2- and 3-ways tagging. The selected TagSNPs provided substantial coverage of all reported SNPs in six of the ten selected genes: *GSK3B*: 74 tagged of 106 total SNPs; *HK1*: 71 tagged of 154 total SNPs; *MAPK14*: 65 tagged of 72 total SNPs; *PCK2*: 5 tagged of 5 total SNPs; *PCK1*: 9 tagged of 16 total SNPs; *FBP1*: 35 tagged of 38 total SNPs. No or limited HapMap data were available for remaining four genes (ENO1, ENO2, GAPDH and PGAM1). Instead, all SNPs reported in dbSNP (NCBI) with a minor allele frequency (MAF) > 0.01 in European populations were chosen for these genes.

### 2.4 Genotyping

Genomic DNA was extracted from whole blood samples. Genotyping was performed using the GoldenGate assay (Illumina Inc)[57] on custom designed Illumina high-throughput Bead Arrays at the genotyping facility at Uppsala University, Sweden. An estimated genotype success rate for each SNP, which takes into account both SNP validation status and assay design for the array, put restrictions on the SNP panel that can be tested. Therefore, SNPs less than 60 bp apart can not both be genotyped; when this occurred we selected the SNP with the highest estimated success rate, followed by highest MAF. For tagSNPs selected from HapMap that did not fulfill the assay criteria, additional SNPs within the same LD block were selected instead. In total, we genotyped 185 SNPs across the 10 candidate genes. A call-rate cut-off value equal to 0.90 was used to ensure genotype data with accuracy above 0.98.

### 2.5 Statistics

#### Hardy-Weinberg equilibrium

We tested Hardy-Weinberg (HW) equilibrium using the chi-square exact test implemented in the "GENETICS" package for R on all heterozygous SNP markers in cases and control subjects separately. SNPs in HW disequilibrium in the control were removed from the subsequent analyses, while those in the patient group were not as disequilibrium may arise due to selection of diseased individuals. It may be argued that correction multiple testing should be performed prior to excluding SNPs in HWD. However, we chose a conservative approach and insisted that only SNPs in (nominal) HWE be analyzed. Overall, 185 markers were genotyped across the ten candidate genes. Based on data from the total sample of 2039 persons we found 21 to be monomorphic. Of the remaining SNPs, eight and 13 showed nominal derivation from Hardy-Weinberg proportions in the control and patients group, respectively (P < 0.05). None of these SNPs were deviating in both groups. Markers were exclude if either monomorphic in the total sample or in HW disequilibrium in the control sample-.

#### Population stratification

We tested for stratification by computing F_st _between the three control-populations (Danish, Swedish and Norwegian) across all polymorphic loci in the six examined genes using Arlequin version 3.1[58]. We find no evidence of population stratification between the healthy controls across the six genes assessed together (F_st _= 0.00057) or for each of the genes individually (F_st _[min] = -0.00077 and F_st _[max] = 0.0036).

#### Association tests

Test for allelic and haplotype association were done as a joint analysis of all three samples. In these combined analyses nationality or gender was included as a covariate to adjust for the potential confounding. We chose to correct for these two variables in all initial analyses (despite that F_st _analysis indicated lack of population stratification) as nationality is a proxy not only of the genetic background but also of the differences in the psychiatric health care systems that may impact on which patients are included into the study. Gender was also included as an obligate confounder, as the study specifically focuses on a gender related hypothesis, estrogen. We used a nominal approach with a significance threshold of p < 0.05.

##### Single marker tests of association

For single marker analysis, nominal p-values were obtained using the Cochran-Mantel-Haenszel test for 2 × 2 × k tables in PLINK [59] (v. 0.99 p) in combination with the Breslow-Day test for heterogeneity in the odds ratios for the disease-SNP association between nationalities and gender, respectively.

##### Multi marker tests of association

Haplotype tests of association were performed within LD blocks of each gene using UNPHASED [60,61] (v. 3.0.5). The block structure constructed from HAPMAP data during tagSNP selection was used for FBP1, GSK3B, HK1, MAPK14, PCK1 and PCK2. For ENO1, ENO2, GAPDH and PGAM1 LD blocks were first defined from the genotype data using the default definition in HAPLOVIEW [62]. In either case the LD blocks are constructed based on the method of Gabriel et al. [63] in which a LD block is formed if 95% of each pairwise SNP comparisons are in 'strong LD' (as defined by D prime). Estimated haplotype frequencies <0.01 were set to zero and the options for individual haplotype effect and confounder effects were applied. All marker combinations and all windows sizes were tested within each LD block.

#### Interaction analyses

The Z-score test for difference in pair wise SNP odds ratios between cases and controls implemented in PLINK [59] (v. 0.99 p) were conducted post hoc to test for pair wise interactions of markers, which showed nominal association signals in either allele or haplotype test.

#### Power analyses

Further, the Chi-Square "Goodness of Fit" Test was used to estimate the chi-square difference between the expected and the observed numbers of significant genes. The power calculations were performed using the CATS-Package^ver1.0 ^as derived from R^ver.2.5.1^: .

## Competing interests

The authors declare that they have no competing interests.

## Authors' contributions

LO: Conceived the idea of combining the unbiased data and the estrogen hypothesis. Performed the analysis. Prepared the manuscript. TH: Conceived the idea of combining the unbiased data and using a clinical hypothesis and participated in data analysis and manuscript preparation. KDJ: Validated the diagnoses of the Danish patients cohort. SD, IM: Designing and handling of genotyping. IA, HH: validated the diagnoses in Swedish patients cohort. HU: Handling and validation of healthy Danish controls. ST, AGW: Validation of clinical factors on the Danish patients cohort. EGJ: Principal investigator of the Swedish cohort. Participated in finalizing the manuscript. OAA: Principal investigator of the Norwegian cohort. Participated in finalizing the manuscript. TW: Principal investigator of the Danish cohort. Conceived the idea of combining the unbiased data and a clinical hypothesis and participated manuscript preparation. All authors read and approved the final manuscript.

## Pre-publication history

The pre-publication history for this paper can be accessed here:



## Supplementary Material

Additional file 1 Allele frequency and association results. The table provides MAF, odds ratio CI95 and p-values adjusted for gender and nationality respectively for all tested SNPs. Click here for file
